# Effect of stem cell conditional medium-loading adhesive hydrogel on TGF-β1-induced endometrial stromal cell fibrosis

**DOI:** 10.3389/fbioe.2023.1168136

**Published:** 2023-05-04

**Authors:** Yuan Zhu, Ting Wang, Ming-Jie Bao, Xiao-Hui Qu, Zeng-Ming Li

**Affiliations:** ^1^ JXHC Key Laboratory of Fertility Preservation, Jiangxi Provincial Maternal and Child Health Hospital, Nanchang, Jiangxi, China; ^2^ Department of Reproductive Health, Jiangxi Provincial Maternal and Child Health Hospital, Nanchang, Jiangxi, China; ^3^ Pathology Department, Jiangxi Provincial Maternal and Child Health Hospital, Nanchang, Jiangxi, China

**Keywords:** adhesive hydrogel, stem cell conditional medium, endometrial stromal cell, uterine adhesion, fibrosis

## Abstract

**Introduction:** Uterine adhesion (IUA) is a severe complication that results from uterine operations or uterine infections. Hysteroscopy is considered the gold standard for the diagnosis and treatment of uterine adhesions. Yet, this invasive procedure leads to re-adhesions after hysteroscopic treatment. Hydrogels loading functional additives (e.g., placental mesenchymal stem cells (PC-MSCs)) that can act as physical barriers and promote endometrium regeneration are a good solution. However, traditional hydrogels lack tissue adhesion which makes them unstable under a rapid turnover of the uterus, and PC-MSCs have biosafety risks when used as functional additives.

**Methods:** In this study, we coupled an adhesive hydrogel with a PC-MSCs conditioned medium (CM) to form a hybrid of gel and functional additives (CM/Gel-MA).

**Results and Discussion:** Our experiments show that CM/Gel-MA enhances the activity of endometrial stromal cells (ESCs), promotes cell proliferation, and reduces the expression of α-SMA, collagen I, CTGF, E-cadherin, and IL-6, which helps to reduce the inflammatory response and inhibit fibrosis. We conclude that CM/Gel-MA can more potentially prevent IUA by combining the physical barriers from adhesive hydrogel and functional promotion from CM.

## 1 Introduction

Intrauterine adhesion (IUA), also known as Asherman Syndrome, is one of the prominent reasons for impaired fertility (March 2011; Dreisler and Kjer, 2019), which is caused by both traumatic and non-traumatic factors. The non-traumatic factors are commonly related to intrauterine infections, while the traumatic factors are iatrogenic, such as pregnancy-related curettage (Dreisler and Kjer, 2019; Rein et al., 2011). IUA shows a variety of symptoms such as uterine amenorrhea, menstrual disorders, pelvic pain, recurrent miscarriage, and even infertility, in clinical diagnosis (Dreisler and Kjer, 2019). The present clinical treatment for uterine adhesions is hysteroscopic adhesion release and postoperative balloon placement with hormonal therapy (AAGL Elevating Gynecologic Surgery, 2017). However, the treatment effect is critically correlated with the degree of adhesions. Especially, for patients with moderate and severe adhesions, the prognosis after hysteroscopic treatment is poor, and some re-adhesions form to aggravate the initial adhesions (Benor et al., 2020; Johary et al., 2014). The re-adhesion rate after hysteroscopic therapy for patients with severe IUA was high, with a live birth rate of only 29.1% (Chen et al., 2020). The statistics based on the classification system from the European Hysteroscopy Society depicted 21%–25%, 29.1%, 38.5%, and 41.9% of recurrence for patients with grade 1-2, grade 3, grade 4, and grade 5 adhesions, respectively (Dreisler and Kjer, 2019). Therefore, safer and more effective methods must be discovered to avoid adhesion formation and ensure functional recovery.

The treatment of IUA requires individualized therapy to recover physical structure, restore function, and stimulate stromal cell regeneration. A thorough cure is the regeneration of stromal cells (Rodriguez-Fuentes et al., 2021). Stem cells exhibit some regeneration functions (Wang et al., 2020) and can effectively treat a few chronic diseases. At present, the clinical primary stem cells used are mesenchymal stem cells (MSCs). Researchers have demonstrated that MSCs transplantation effectively relieves intrauterine adhesions (Song et al., 2021). But the transplantation of undifferentiated or improperly differentiated stem cells carries a significant risk of tumorigenesis (Sharpe et al., 2012). Stem cells secrete some active substances (Jabbehdari et al., 2020; Sato et al., 2018) with good curative effects that help in repair function (He et al., 2022; Yamashita et al., 2018). The culture medium containing the secretion of MSCs is named as the conditioned medium (CM). CM can be used to promote cell proliferation and damage repair to avoid the ethical and safety issues of stem cells (Sagaradze et al., 2019). However, CM cannot stay in the uterine cavity for a long time. This is because CM easily flows due to its liquidity when the patient’s position changes. The short duration of CM in the uterine cavity reduces its therapeutic effect. Moreover, the efflux of CM causes symptoms of vaginal discharge and results in discomfort and vaginitis.

The application of hydrogels to biomedical research is a hot topic. Hydrogels possess good three-dimensional morphology, controlled swelling, biodegradation properties, and excellent cytocompatibility. They can also be modified for additional properties (Tytgat et al., 2019). They are widely used as wound dressings and biological scaffolds to promote tissue regeneration and injury repair (Colangelo et al., 2021; Hu et al., 2023; Tytgat et al., 2019; Wen et al., 2022) because of their superb adjustability. Researchers have found that some hydrogel-derived biological scaffolds not only support endometrial cell attachment and proliferation, but also deliver stem cells or bioactive molecules that promote endometrial regeneration (Li et al., 2021). A CS/Exos structure with a collagen scaffold and UC-MSCS-derived exosomes promotes endometrial regeneration and fertility recovery through macrophage immunomodulation (Xin et al., 2020). However, because of the rapid turnover of the uterus, these gels cannot stay stable when applied. A healthy uterus of women secretes 3-4 g of endometrial mucus every 4 h (Ensign et al., 2012). Hydrogels with adhesion properties can perfectly solve this problem. After application, the adhesion hydrogels can easily stay at the applied surface because of the interaction with tissues. Methacrylate-modified gelatin (Gel-MA) is a biodegradable polymer that can easily form adhesive hydrogels with photo initiation (Liu and Chan-Park, 2010). Gel-MA-based adhesive hydrogels are good scaffolds for wound healing (Stubbe et al., 2021), cartilage regeneration (Shi et al., 2021), and dental repair (Wang et al., 2021) with good adhesion properties (Pirmoradian et al., 2022). Moreover, it can easily load functional additives (Lim et al., 2019). Although the use of Gel-MA adhesive hydrogels in IUA prevention is very promising, few studies have been reported.

**FIGURE 1:**
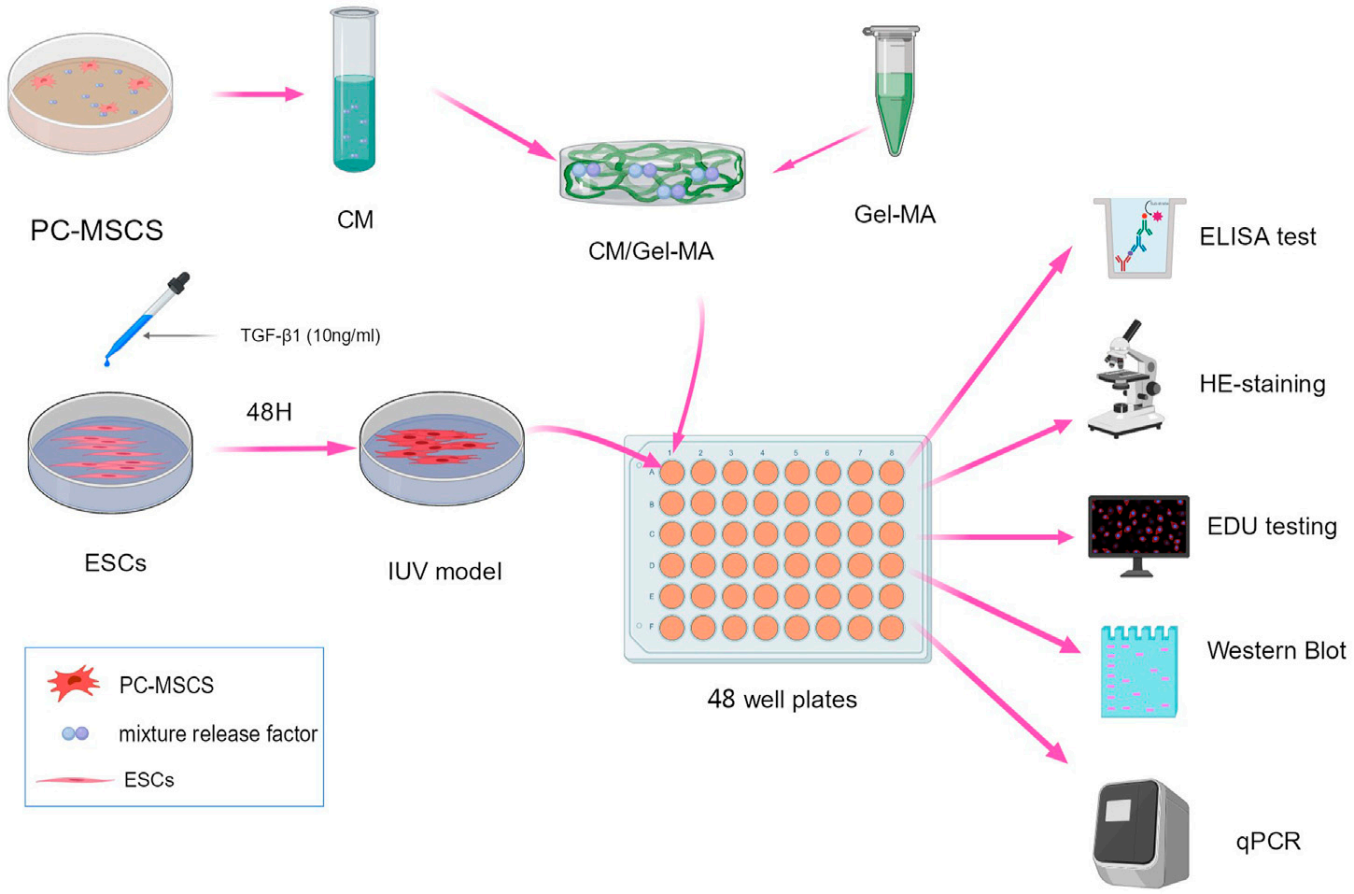
Scheme of the experimental procedure. All schematics are drawn using MedPeer (www.medpeer.com).

In this study, we designed a CM-loaded adhesive hydrogel (CM/Gel-MA) based on Gel-MA. CM/Gel-MA is greatly adhesive which favors the stable retention of CM. The potential anti-IUA effect of CM/Gel-MA has been tested on the classical uterine adhesion cell model (Figure 1). CM/Gel-MA was found to improve cell viability, promote cell proliferation, and significantly reduce fibrosis, implying its potential for cell adhesion inhibition. This suggests that Gel-MA is a suitable biological scaffold in IUA injury repair and CM/Gel-MA is a potential agent for IUA treatment.

## 2 Materials and methods

### 2.1 Experimental materials

TGF-β1 (PEPRO TECH, China, 100-21-10 μg), PC-MSC (Provided by Shenzhen 150 Biomedical Co.,Ltd.), ESC (Provided by ATCC), FITC Mouse IgG1 κ Isotype control (Biolegend, 7313762), PE Mouse IgG1 κ Isotype control (Biolegend, 9217868), APC Mouse IgG1 κ Isotype control (Biolegend, 9282592), PE Mouse Anti-Human CD73 (Biolegend, 8151901), APC Mouse Anti-Human CD105 (Biolegend, 9277133), FITC Mouse IgG2a κ Isotype control (Biolegend, 8241935), FITC Mouse Anti-Human CD14 (Biolegend, 8299630), FITC Mouse Anti-Human CD45 (Biolegend, 8206704), DMEM/F12 medium (Peiyuan, Shanghai, L310 kJ), Fetal bovine serum (Gemini, 900-108), Trypsin digestive fluid (Beyo, C0203), DPBS (Beyo, C0221G), CCK8 (Beyotime, C0039), EDU staining kit (Beyotime, C0081s), Hematoxylin-eosin staining kit (Solarbio, G1120), DEPC-treated water (Solarbio, R1600), trichloromethane (Sinopharm Chemical Reagent Co. LTD., 10006818), GelRed dye (Biotium, #41003), RNA Loading Buffer (TaKaRa, 9168), Tris-Borate-EDTA Buffer (TBE) 10× Powder, pH8.3 (TaKaRa, T9122), RNAsimple Total RNA Kit (TIANGEN, DP419), All-in-One First-Strand Synthesis Master Mix (Yugong Biolabs, EG15133S), SuperReal PreMix Plus (SYBR Green) (TIANGEN, FP205-02), and Methacrylate anhydride (MAA, 0.1 mL/1 g gelatin), ELISA Kit (Solarbio, SEKH-0052), MateRegen^®^ Gel (2104006, BioRegen Biomedical (Changzhou) Co., Ltd.).

### 2.2 Methods

#### 2.2.1 Establishment of the IUA model

Endometrial stromal cells (ESCs) were first cultured with 80–90% confluence. Then, cells were digested, collected, and dispersed into 6-well cell culture plates with a density of 1.0×10^5^ cells/mL for further incubation at 37°C, in a 5% CO_2_ cell culture incubator (Thermo Fisher Scientific, Heracell 240i). When the confluence reached approximately 80%, TGF-β1 (10 ng/mL) was added to the plate for another 48 h treatment to induce the IUA model (Liu et al., 2020).

#### 2.2.2 Synthesis of Gel-MA

Gel-MA was prepared according to a previous report (Zhu M. et al., 2019). Gelatin (10 g) was dissolved at 10 (w/v) % in a carbonate–bicarbonate (CB) buffer solution (0.25 M, 100 mL) and heated to 50°C. Methacrylate anhydride (MAA, 0.1 mL/1 g gelatin) was added to the fully dissolved gelatin solution at 500 rpm magnetic stirring, and the reaction pH was adjusted to 9. After 3 h reaction at 50°C, the solution was adjusted to pH 7.4 to stop the reaction. The final solution was purified by dialysis in an aqueous solution at 50°C. Finally, Gel-MA solid products were obtained by freeze-drying.

#### 2.2.3 Acquisition of CM

PC-MSCS (Provided by Shenzhen 150 Life Technology Co., LTD.) were cultured (Source Biologicals, T310JV) and continuously passaged until P3 generation PC-MSC cells were collected. Subsequently, cells were identified by positive (PE-CD73, APC-CD105) and negative (FITC-CD14, FITC-CD45) biomarkers. After confirming the phenotype of the cells, they were continuously cultured to P5 generation. The collected cells were incubated with a serum-free DMEM/F12 (Shanghai Peiyuan, L310 kJ) medium, and were continuously cultivated for 48 h. Finally, the CM supernatant obtained was collected for further use.

#### 2.2.4 *In vitro* lap shear test of Gel-MA and CM/Gel-MA

The shear resistance of Gel-MA (*n* = 3) and CM/Gel-MA (*n* = 3) was tested following the previous report (Annabi et al., 2017) with some modifications. Each sample was examined using two glass slides (25 mm and 75 mm) with the top (25 mm and 10 mm) coated with gelatin solution. At room temperature, gelatin-coated glass slides were dried. The Gel-MA solution (40 L) and the CM/Gel-MA solution (40 L) were then added to one of the gelatin-coated areas, and the other slide was placed in the solution. Subsequently, UV light irradiation was used to cross-link the hydrogel between the two glass slides. Finally, the shear strength of the hydrogel was determined using an electronic universal testing equipment (CMT1103, Zhuhai Sansi Test Equipment Co., Ltd., China).

#### 2.2.5 *In vitro* wound closure test of Gel-MA and CM/Gel-MA

An *in vitro* wound closure test was conducted in accordance with the grouping of the shear experiment. The test was carried out using sliced, rectangular-shaped pig skin (length 20 mm, width 10 mm). The pig skin was then split along the middle using a knife to imitate an injury. Gel-MA (10 L) and CM/Gel-MA (10 L) solutions were injected into both sides of the wound. The hydrogels were then individually photo-crosslinked on both surfaces using UV light irradiation. Finally, an electronic universal testing machine was used to assess the wound closure strength (CMT1103, Zhuhai Sansi Test Equipment Co., Ltd., China).

#### 2.2.6 Adhesion test

The test was carried out using sliced, rectangular-shaped pig skin (length 30 mm, width 10 mm). To begin, 100 μL of Gel-MA, CM/Gel-MA, and hyaluronic acid hydrogels (HA) (MateRegen^®^ Gel (2104006, BioRegen Biomedical (Changzhou) Co., Ltd.) were applied to rectangular pig skin. The Gel-MA and CM/Gel-MA are then illuminated with UV light. Finally, the gel was rinsed using PBS with a 5 mL pipette at an even rate.

#### 2.2.7 ELISA analysis

The slow release of CM/Gel-MA was investigated by measuring the VEGF content of CM (*n* = 3). Following the manufacturer’s instructions, VEGF levels were measured using an ELISA kit (Solarbio, SEKH-0052).

#### 2.2.8 CCK-8 assays for cell proliferation evaluation

ESCs after TGF-β1 treatment were collected, digested, and made into a single cell suspension at 1×10^4^ cells/well (edge wells are filled with sterile PBS to eliminate edge effect). The cells were then incubated for approximately 6 h. After adhering to the plate, Gel and control agents were added. After another 48 h incubation, 250 μL of CCK-8 solution (Beyotime, C0039) was added to each well and again incubated for 2 h. Finally, the OD values at 450 nm were read and recorded by a plate reader (Biotek, Elx808).

#### 2.2.9 H&E staining for pathological check

Each sample was washed with PBS 1–2 times. Then, 200 μL of paraformaldehyde (4%) was added to the samples and incubated for 30 min at room temperature. Subsequently, 200 μL of hematoxylin staining solution (Solebro, G1120) was added for 25 min incubation at room temperature, followed by 200 μL eosin incubation (Solaibao, G1120) for 1 min. Finally, the stained samples were washed and immediately observed.

#### 2.2.10 EDU assay for cell proliferative capacity evaluation

EDU (Beyotime, C0081s) was first diluted into a DMEM/F12 cell medium at a ratio of 250:1 to reach a concentration level of 40 μM. Then, 100 μL of 40 μM EDU medium was added to each well and incubated for 24 h. After washing with PBS, 200 μL of paraformaldehyde (4%) was added to fix them. Then, they were decolorized and shaken for 5 min, followed by a sequential addition of 200 μL permeabilizer (0.3% Triton-100) and 200 μL 1× click reaction solution. After another 30 min incubation, 100 μL of 1× Hoechst 33342 reaction solution was added to each well and incubated for 10 min at room temperature. After washing with PBS, using the 405 nm channel of a fluorescence microscope, it was observed and photographed.

#### 2.2.11 mRNA level evaluation of α-SMA, collagen I, CTGF, E-cadherin, and IL-6

Total RNA was extracted from cells with an RNAsimple total RNA kit (TIANGEN, DP419), and was stored at −80°C. To quantify the mRNA level of biofactors, the corresponding primer pairs were designed and synthesized, as listed in Table 1. Hsa-β-actin was chosen as the internal reference primer for PCR, whose forward and back sequences were TTC​CTT​CCT​GGG​CAT​GGA​GTC and TCT​TCA​TTG​TGC​TGG​GTG​CC, respectively.

**TABLE 1:** Primers utilized in quantitative real-time PCR.

Primer name	Forward primer	Reverse primer
α-SMA	CGT​TAC​TAC​TGC​TGA​GCG​TG	TGA​AGG​ATG​GCT​GGA​ACA​GG
collagen I	GGA​CAC​AGA​GGT​TTC​AGT​GG	CAG​TAG​CAC​CAT​CAT​TTC​CAC​G
CTGF	CTG​GTC​CAG​ACC​ACA​GAG​TG	TGC​CCT​TCT​TAA​TGT​TCT​CTT​CCA
E-cadherin	GCT​GGA​CCG​AGA​GAG​TTT​CC	CGA​CGT​TAG​CCT​CGT​TCT​CA
IL-6	ACT​CAC​CTC​TTC​AGA​ACG​AAT​TG	CCA​TCT​TTG​GAA​GGT​TCA​GGT​TG

#### 2.2.12 Western blot (WB) assay

To confirm the protein expression of interest, western blot was carried out as previously descrbied (Ai et al., 2020). The primary antibodies used were listed in Table 2. Amount of the protein of interest was normalized to the densitometric units of β-actin.

**TABLE 2:** Antibodies.

Antibody	Source	Cat. no	Brand	Dilution
β-Actin	Rabbit	20536-1-AP	Proteintech	1:1000
α-SMA	Rabbit	14395-1-AP	Proteintech	1:1000
collagen I	Rabbit	14695-1-AP	Proteintech	1:1000
CTGF	Rabbit	25474-1-AP	Proteintech	1:1000
E-cadherin	Rabbit	20874-1-AP	Proteintech	1:20000
IL-6	Rabbit	21865-1-AP	Proteintech	1:500

#### 2.2.13 Statistical analysis

All experimental results mentioned in this study are expressed as the mean ± SD. Statistical analysis was carried out using GraphPad Prism 9.0 software (GraphPad Software, San Diego, CA, United States) and Origin software (Origin Lab, United States). The *t*-test was used to compare two conditions. ANOVA with a Bonferroni post-test was used for multiple comparisons. The *p*-value <0.05 was considered statistically significant.

## 3 Results and discussion

### 3.1 Preparation and characterization for CM/Gel-MA

**FIGURE 2:**
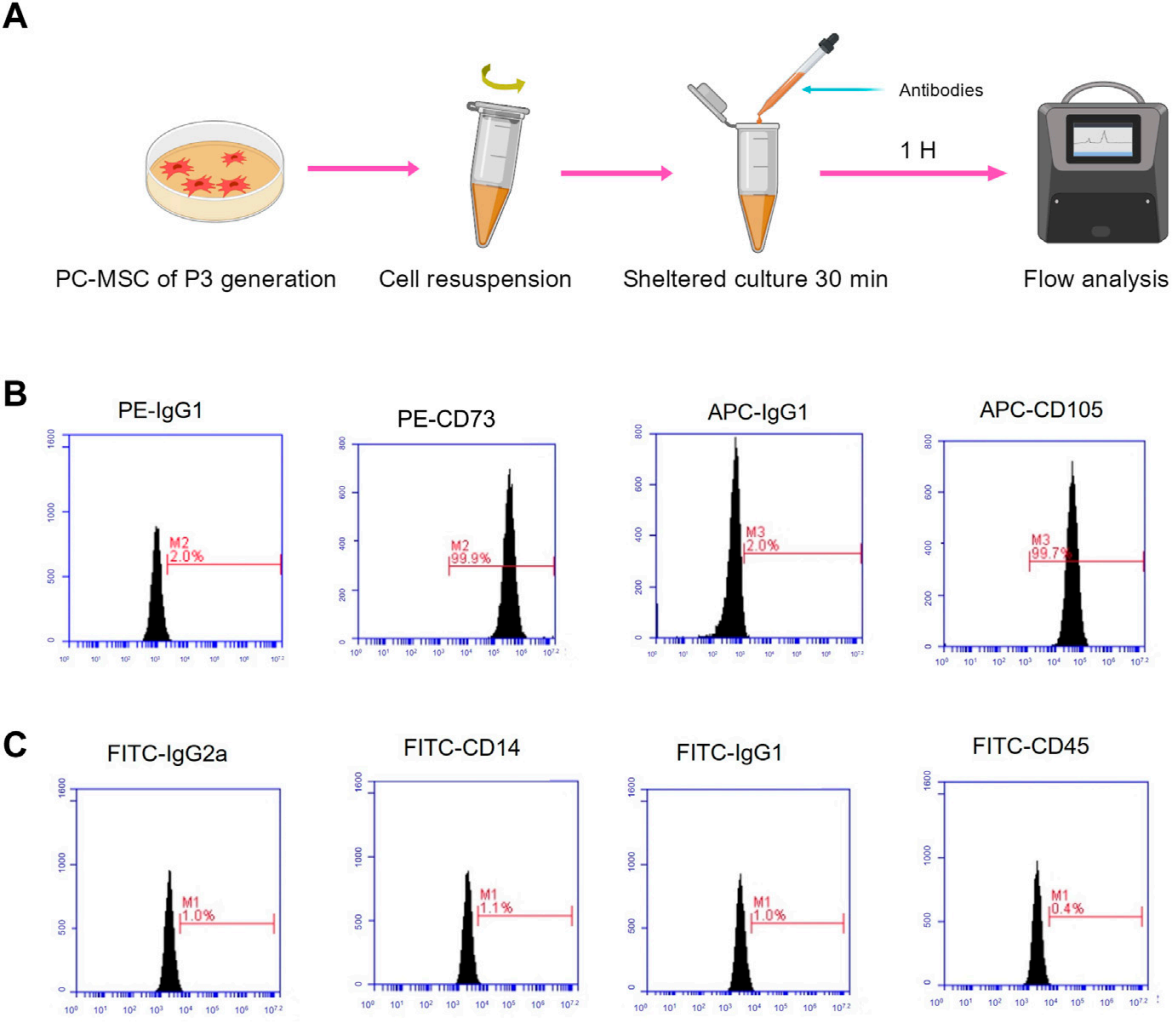
PC-MSC immunophenotype identification. **(A)** Schematic diagram of flow analysis. **(B)** Test for CD73 and CD105 expressions. **(C)** Test for CD14 and CD45 expressions.

Before preparing the CM, the PC-MSC phenotype was strictly identified. We followed the regular procedure and identified PC-MSCs by flow cytometry (Figure 2A). As shown in Figures 2B, C, the expression of CD73 and CD105 was 99.9%, and 99.7%, respectively, whereas both CD14 and CD45 were expressed at low levels (less than 5%). These results indicated that the cells used were PC-MSCs.

**FIGURE 3:**
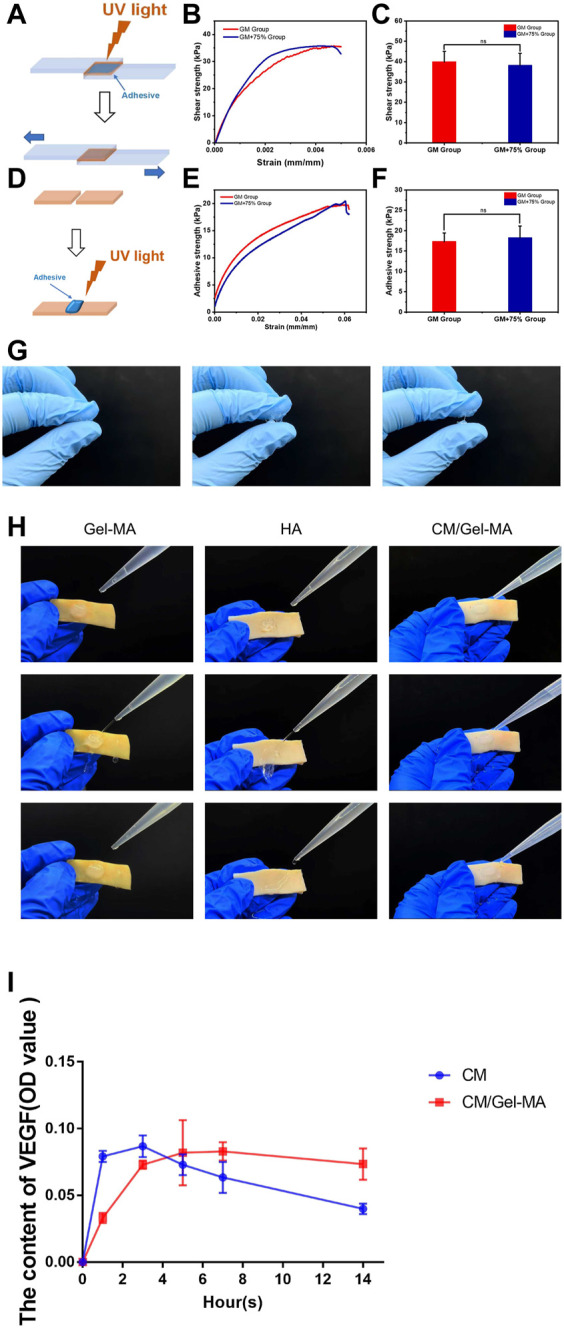
Evaluation of gel properties. **(A** and **D)** Schemes showing the lap shear test (A) and wound closure test of Gel-MA and CM/Gel-MA. **(B** and **C)** The shear strength of Gel-MA and CM/Gel-MA (*n* = 3, mean ± SD, ns: not significant). **(E** and **F)** The wound closure strength of Gel-MA and CM/Gel-MA (*n* = 3, mean ± SD, ns: not significant). Statistical significance calculated using Student’s *t*-test is given in **C** and **F**. **(G)** The tensile resistance of HA. **(H)** Adhesion test. **(I)** The results of the ELISA. CM/Gel-MA group: Gel containing 75% CM (100 μL) + PBS(800 μL); CM group: 75% CM solution (100 μL) + PBS (800 μL).

Following a previous report, CM concentrations of 75% were chosen to fabricate CM/Gel-MA for performance evaluation (Zhu H. et al., 2019). Appropriate adhesion favors the retention of hydrogel and increases the duration of loaded factors in the uterus. Gels from Gel-MA have good tissue adhesion (Pirmoradian et al., 2022). However, the influence of CM on Gel-MA’s adhesion performance is unknown. As a result, the adhesion strength of Gel-MA and CM/Gel-MA was evaluated by a lap shear test (ASTM F2255-05) and a wound closure test (ASTM F2458-05). The lap shear tests in Figures 3A–C demonstrate that the lap shear strength of the Gel-MA group is nearly the same as that of the CM/Gel-MA group, without a statistical difference. It indicated that the addition of CM did not change the adhesion performance much. The wound closure tests were carried out further (Figures 3D–F). It still revealed that the wound closure strength of the Gel-MA group was 17.29 ± 2.14 kPa, which was not statistically different from that of CM/Gel-MA (18.23 ± 2.92 kPa). Hence, the addition of PMCSCM to Gel-MA did not significantly reduce the adhesion performance of Gel-MA.

### 3.2 CM/Gel-MA has better adhesion effect than HA and can gradually release CM

HA, like a gelatin, is now widely used in tissue regeneration and wound healing (Bonafe et al., 2014; Rao et al., 2023). HA can also be used as a transporter in the treatment of intrauterine adhesions (Liu et al., 2019). However the advantage of gelatin over hyaluronic acid is that gelatin can offer tissue adhesiveness through ionic bonding with tissue (Duan et al., 2023; Bu and Pandit, 2022). Here, the commercially available HA hydrogel of IUA prevention was used as control. Figure 3G showed that HA hydrogel didn’t show any sign of adhesion. As a result, lap shear and wound closure experiments can’t be carried out. To further show the different performance in adhesion, the Gel-MA hydrogels and HA hydrogels were applied on the pig skin. Figure 3H showed that Gel-MA and CM/Gel-MA adhered firmly to pig skin even under water flow while HA hydrogel was quickly washed away. A good tissue adhesion can increase the stability of those functional hydrogels, thus increasing the therapeutic efficacy (Feng et al., 2023).

CM has a complex composition, and studies have revealed that it contains a variety of cytokines of which VEGF is one (Zagoura et al., 2012; Pawitan, 2014). Here to show that after loading CM into Gel-MA, the system can gradually release the bioactive components, VEGF was used as the representive and the release was detected by ELISA. Figure 3I showed that after dissolving CM in the medium, the content of VEGF reached maximum and gradually decreased. The decreased amount might result from the denaturation of VEFG. However, a gradually increased amount of CM was detected in CM/Gel-MA system, which kept stable even after 14 h of release. These experiments showed that after loading CM into Gel-MA, the system achieved a gradually release of CM and might even help prevent the VEGF from denaturation.

### 3.3 CM/Gel-MA attenuates fibrosis and promotes the proliferation of ESCs

**FIGURE 4:**
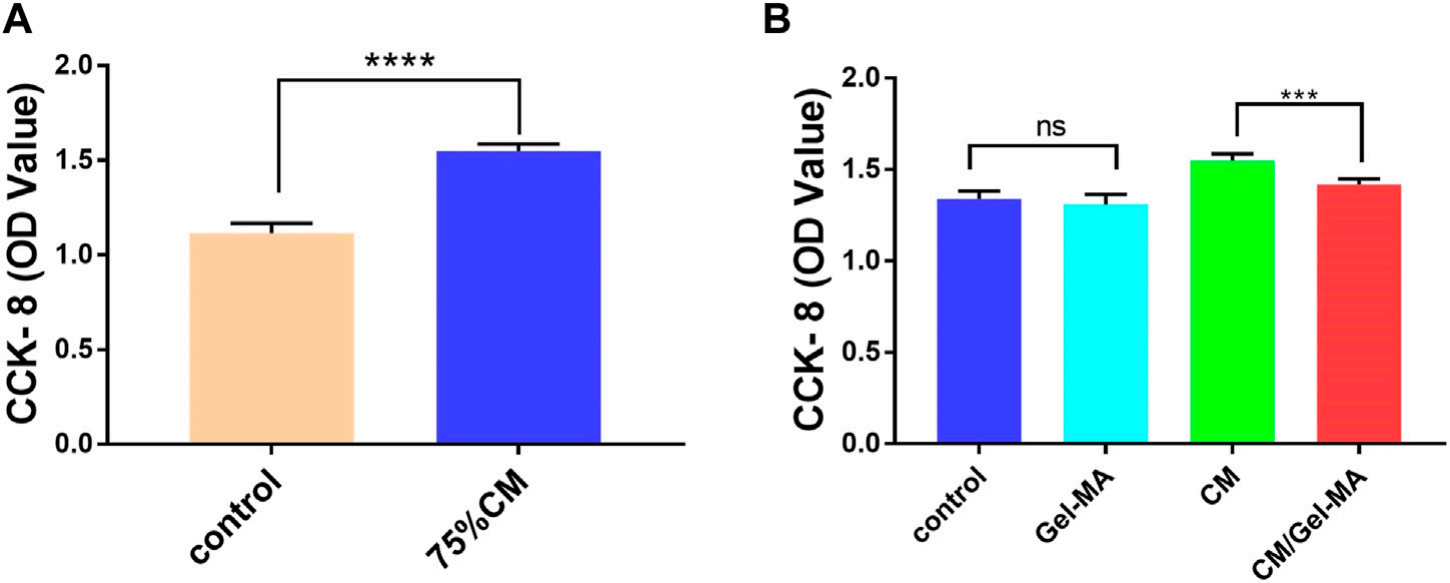
CCK-8 assays for cell proliferation. **(A)** 75% CM promotes cell proliferation. Control group: Complete medium (800 μL) + serum-free medium (100 μL); 75% CM group: 75% fresh CM (800 μL) +75% serum-free CM medium (100 μL); **(B)** Proliferation ability of ESCs in different media. All subgroups contain 800 μL of 75% fresh CM as the basic medium. Control group: serum-free medium (100 μL); Gel-MA group: Gel containing serum-free medium (100 μL); CM group: 75% serum-free CM medium (100 μL); CM/Gel-MA group: Gel containing 75% serum-free CM (100 μL). (***: *p* < 0.001; **: *p* < 0.01; *: *p* < 0.01; ns: >0.05).

ESCs were closely associated with IUA treatment. As ESCs increased, IUA levels decreased. Thus, we assessed the apparent number of ESCs treated with CM, Gel-MA, or CM/Gel-MA in the IUA model using the CCK-8 assay. The OD value increased as the apparent number of cells increased. First, compared with the control group, there was a significant enhancement in the relative cell numbers of the group with 75% CM treatment (Figure 4A). This indicated that CM addition improved cell proliferation, which was consistent with the previous results (Wei et al., 2022; Zhu H. et al., 2019). Then, we investigated the effect of Gel-MA on ESCs proliferation. No significant difference was observed in cell numbers between the groups with or without Gel-MA treatment. This means Gel-MA is a suitable material for safe application to the endometrium. It was also observed that cells treated with CM/Gel-MA had fewer cell numbers than those treated with CM of the same amount, which is a significant difference (Figure 4B). This is because slowly released CM in Gel-MA could not perform the highest promotion effect in a short time. However, regular therapy needs a longer period to match with the sustained release system. Hence, CM/Gel-MA can be used for long-term therapy.

**FIGURE 5:**
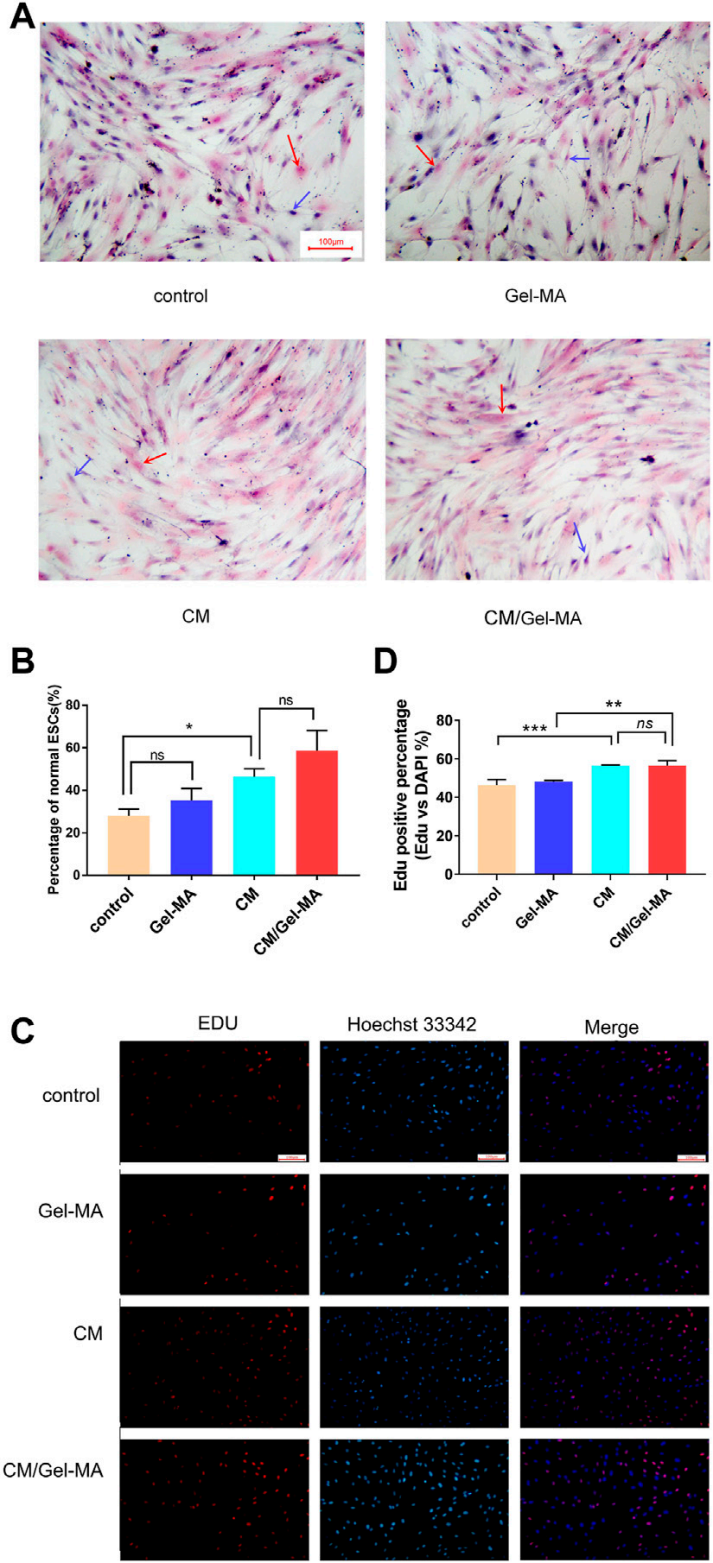
CM/Gel-MA enhances cell viability and reduces fibrosis. **(A)** H&E staining (100×). The nucleus is stained blue and the cytoplasm is stained pink. Blue arrows indicate normal ESC and red arrows indicate post-fibrotic ESC. **(B)** Percentage of normal ESCs. **(C)** EDU assay for cell viability, cells in the proliferative phase show strong red fluorescence after EDU staining and blue fluorescence reaction of the nucleus after Hoechst 33324 staining. **(D)** Statistical analysis table of EDU assay for cell viability. (***: *p* < 0.001; **: *p* < 0.01; *: *p* < 0.01; ns:>0.05).

We then investigated the ESCs proliferation state and evaluated CM/Gel-MA mediated ESCs fibrosis by H&E staining, after different treatments. ESCs are adnexal cells with a morphology similar to fibroblasts, with a long spindle shape and a large nucleus (Queckborner et al., 2020). In Figure 5A, after TGF-β1 treatment, the morphology of some ESCs changed: cytoplasm, nucleus, and multiple branches on the cell membrane faded, and the original spindle shape was lost. This indicated the occurrence of fibrosis in ESCs. Whereas, after CM or CM/Gel-MA treatment, most cells recovered their original long shuttle shape. We also counted the percentage of normal ESCs in each group, and the results revealed that the percentage of normal ESCs increased after CM or CM/Gel-MA treatment (Figure 5B). Both CM and CM/Gel-MA attenuated fibrosis.

EDU (5-ethynyl-2′deoxyuridine nucleoside) is a thymidine nucleoside analog, which can light on cells in the proliferative phase. As shown in Figures 5C, D, after being stained by EDU, more ESCs are observed in a proliferating state in the groups treated by CM and CM/Gel-MA. These results are consistent with those from Wei’s study, which demonstrated the positive effect of CM on IUA (Wei et al., 2022). There was also no significant difference between CM and CM/Gel-MA, which means Gel-MA did not hinder the CM’s function of cell growth promotion. In addition, ESCs treated by Gel-MA showed a similar activity with the control group. It solidly implied that Gel-MA was biocompatible. Overall, our result confirmed that CM/Gel-MA significantly promotes ESC proliferation like CM, and also, Gel-MA is safe for use and potentially synergistic for CM application.

### 3.4 CM/Gel-MA reduces the expression of some pro-fibrotic factors, pro-adhesion molecules, and attenuates fibrosis

**FIGURE 6:**
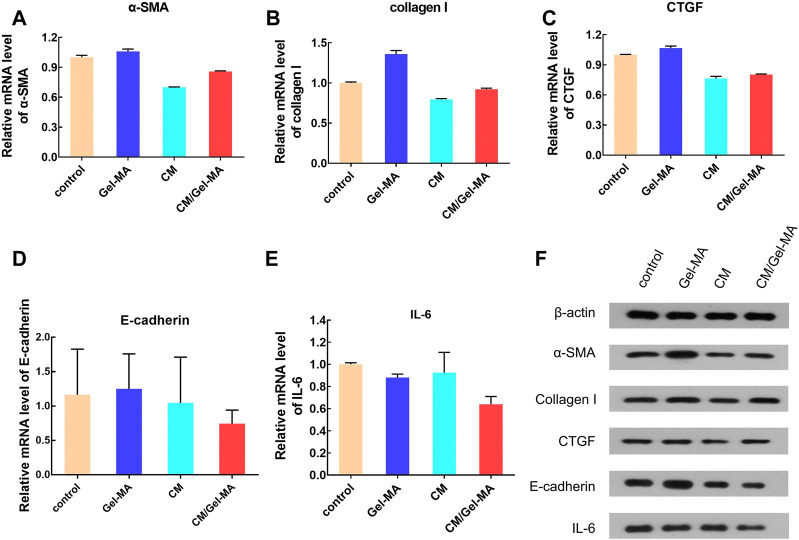
Expression level of some bio-factors. **(A)** Relative mRNA level of α-SMA. **(B)** Relative mRNA level of collagen I. **(C)** Relative mRNA level of CTGF. **(D)** Relative mRNA level of E-cadherin. **(E)** Relative mRNA level of IL-6. **(F)** The results of WB.

The abnormality of the TGF-β/Smad signaling pathway plays an important role in fibrosis. Through Smad2 or Smad3, the α-SMA level can be upregulated, thus promoting fibrosis (Zeisberg and Kalluri, 2013). In accordance with previous reports, α-SMA would be significantly elevated in ESCs after TGF-β1 treatment (Li et al., 2016; Zhu H. et al., 2019). Therefore, it was used as a control group to mimic disease status to investigate CM/Gel-MA’s effect. As shown in Figure 6A, the mRNA expression of α-SMA was slightly enhanced by the addition of Gel-MA in the IUA model, while it decreased after being treated with CM. When using CM/Gel-MA, downregulation of mRNA expression is still observed, but the degree of attenuation is less than that of the CM-only group. CM/Gel-MA is advantageous because CM is gradually released and has a durable effect with the degradation of Gel-MA in CM/Gel-MA.

Collagen I is a fibrogenic factor mainly found in the cell stroma (Li et al., 2016). The expression of collagen I significantly increases with fibrosis in endometrial cells (Sun et al., 2019). Our study revealed that CM decreases the mRNA expression of collagen I (Figure 6B), consistent with the previous reports (Yu et al., 2018). Importantly, CM/Gel-MA also downregulated collagen I mRNA expression (Figure 6B). However, the slow-release effect partially decreased CM function in a limited treatment period. This result still indicated the potential applicability of CM/Gel-MA against fibrosis. In addition, the expression of CTGF, another indicator evaluating fibrosis, was tested. CTGF expression increased when IUA occurred (Chen et al., 2021), but it could be significantly decreased by CM or CM/Gel-MA treatment (Figure 6C). In summary, CM/Gel-MA can downregulate the mRNA expression of α-SMA, collagen I, and CTGF, which is its function mechanism to inhibit ESC fibrosis.

E-cadherin is an important factor involved in the intercellular adhesion and maintenance of cytoskeletal integrity (Gumbiner, 2005; Wynn, 2007). Epithelial-mesenchymal transformation (EMT) has been shown to be associated with fibrosis (Marconi et al., 2021), while E-cadherini is essential for EMT (Bure et al., 2019), so E-cadherin may be associated with fibrosis. However, compared with the control group, the mRNA level of E-cadherin is decreased when treated with CM or CM/Gel-MA, and CM/Gel-MA downregulated E-cadherin better (Figure 6D). Therefore, CM/Gel-MA effectively downregulated mRNA expression of E-cadherin and might prevent endometrial fibrosis. Moreover, both CM and CM/Gel-MA decreased IL-6 expression (Figure 6E), and the effect of CM/Gel-MA was more pronounced. It indicated reduced inflammation which helped fibrosis inhibition (Vahedian et al., 2020). Therefore, the downregulation of E-cadherin and IL-6 mRNA levels supported the conclusion that CM/Gel-MA had the potential to attenuate IUA fibrosis.

**FIGURE 7:**
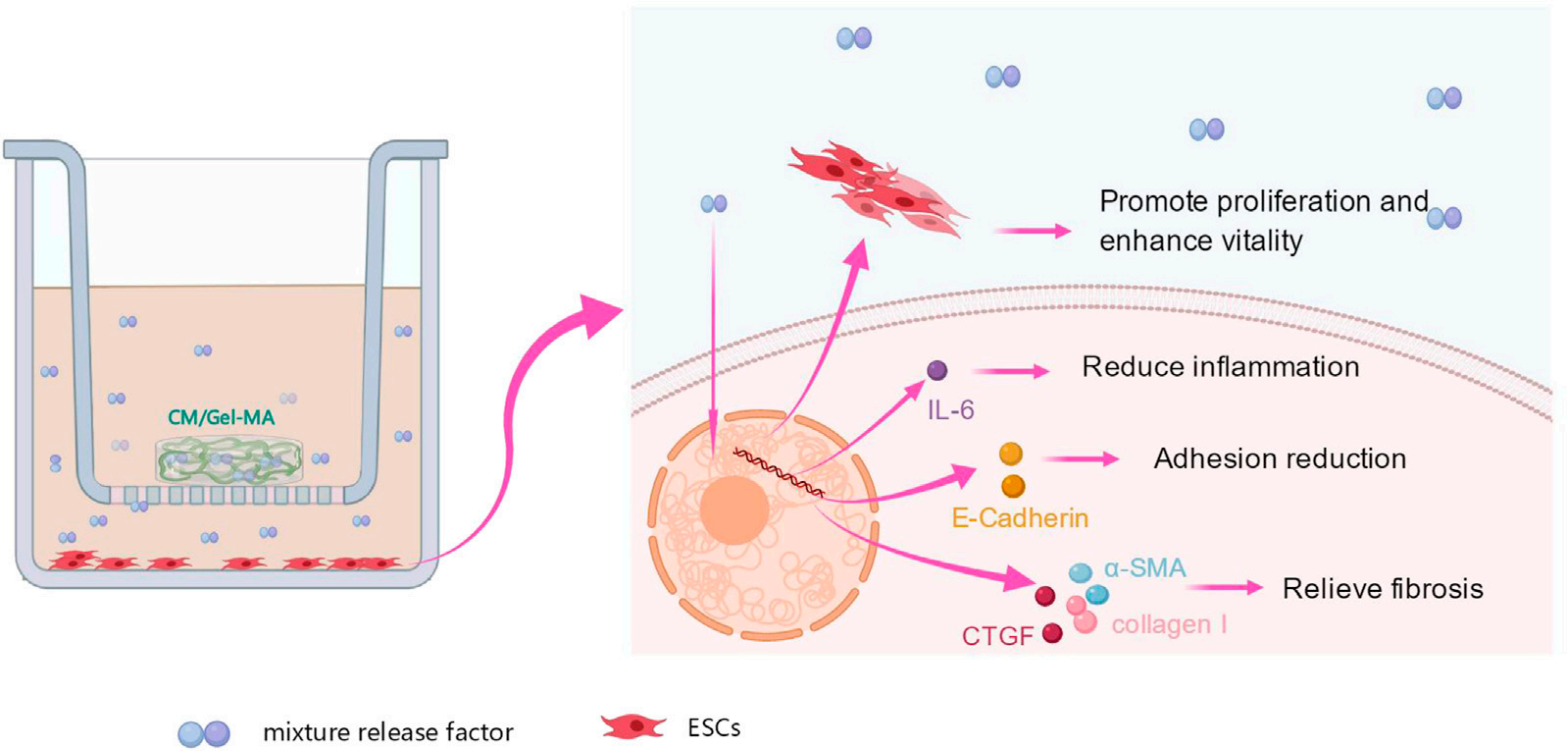
Effect of CM/Gel-MA on IUA cell model. The IUA model was induced by adding TGF-β1 into endometrial stromal cells. After treating the model with CM/Gel-MA, the fibrosis was inhibited with decreased the expression of α-SMA, collagen I, CTGF, E-cadherin, and IL-6. At the same time, the cell vitality and proliferation ability were enhanced after treatment.

Further, the WB experiments were carried out to show the expression of the fibrosis-associated proteins. Figure 6F showed that all trends of those protein expression were basically consistent with the results of qPCR. As a result of the aforementioned findings, it is summarized that CM/Gel-MA reduces the expression of α-SMA, collagen I, CTGF, E-cadherin, and IL-6. Good CM functions as well as Gel adhesion on the uterine cavity may help CM/Gel-MA to have a long-term curation effect on IUA (Figure 7).

## 4 Conclusion

In summary, we explore a new method of Gel-MA-assisted CM for uterine adhesion relief. A simple and environmentally friendly procedure is used to create the CM/Gel-MA hybrid. In this hybrid, CM is used to regulate the levels of some biofactors and resist uterine adhesion, whereas Gel-MA is used as a supporter with great adhesive capability to fix CM at the uterine wall and slowly release it. CM addition does not weaken the mechanical properties of Gel-MA. The CM/Gel-MA scaffold efficiently executes CM functions, such as increasing cell survival and proliferation, inhibiting the expression of α-SMA, collagen I, CTGF, E-cadherin, and IL-6, and thereby preventing fibrosis and inflammation. In the next phase, we will conduct experimental studies from the animal level to further explore CM/Gel-MA in IUA.

## Data Availability

The datasets presented in this study can be found in online repositories. The names of the repository/repositories and accession number(s) can be found in the article/supplementary material.
